# Association between Periodontal Disease and Comorbidities in Saudi's Eastern Province

**DOI:** 10.1155/2021/5518195

**Published:** 2021-04-16

**Authors:** Marwa Madi, Hatem M. Abuohashish, Dina Attia, Norah AlQahtani, Nabras Alrayes, Verica Pavlic, Subraya G. Bhat

**Affiliations:** ^1^Department of Preventive Dental Sciences, College of Dentistry, Imam Abdulrahman Bin Faisal University, Dammam 31411, P.O. Box 1982, Saudi Arabia; ^2^Department of Biomedical Dental Sciences, College of Dentistry, Imam Abdulrahman Bin Faisal University, Dammam 31411, P.O. Box 1982, Saudi Arabia; ^3^Department of Pediatric Dentistry and Dental Public Health, Faculty of Dentistry, Alexandria University, Alexandria 21527, Egypt; ^4^College of Dentistry, Imam Abdulrahman Bin Faisal University, Dammam 31411, P.O. Box 1982, Saudi Arabia; ^5^Department of Periodontology and Oral Medicine, Institute of Dentistry, Banja Luka 78000, Bosnia and Herzegovina; ^6^Department of Periodontology and Oral Medicine, Medical Faculty University of Banja Luka, Banja Luka 78000, Bosnia and Herzegovina

## Abstract

The incidence of periodontal diseases is associated with multiple comorbidities that influence a patient's treatment planning. This study evaluates the relation between periodontal disease and multiple comorbidities reported in the Saudi population from the Eastern province. This study was conducted on 190 patients, who visited the periodontology clinics at Imam Abdulrahman Bin Faisal University, Saudi Arabia. Demographic data, smoking habits, past medical and dental histories, blood pressure, random blood glucose, and recent haemoglobin A1c were recorded. A comprehensive periodontal examination included the number of missing teeth, pocket depth (PD), clinical attachment level (CAL), bleeding on probing (BOP), and mobility of all teeth except third molars. Radiographic bone loss was measured on standardized full-mouth periapical radiographs. Multivariable regression models were calculated aiming to see the association between different comorbidities and alveolar bone loss with confounders controlled. Out of 190 periodontitis patients, 56 (29.5%) were males and 134 (70.5%) were females. More than half of the patients (60%) were between 26 and 50 years, 30% of them had diabetes, and 18% were smokers. The risk of alveolar bone loss was higher in persons who had diabetes and those who had both diabetes and coronary heart disease than those who did not, although the association was not statistically significant (*B* = 1.26, 95%CI = −0.30, 2.82, and *B* = 2.86, 95%CI = −1.25, 6.96, respectively). The risk of alveolar bone loss was significantly higher among persons with diabetes and hypertension (*B* = 2.82 and 95%CI = 0.89, 4.75). Collectively, the risk of alveolar bone loss in periodontitis patients increases with diabetes in the presence of other comorbidities regardless of smoking or gender.

## 1. Introduction

Periodontal diseases comprise periodontitis and gingivitis, which are responsible for the destruction of the supporting tissues of the tooth apparatus and are the major cause for loosing teeth among adults [1]. Periodontal diseases are common, and their prevalence varies in different populations including adolescents, adults, and older individuals, which might represent a public health concern [2]. At the age of 40, the prevalence of severe periodontitis peaks and then remains stable in older ages [3]. It is important to highlight that the prevalence of periodontal disease will be increasing in the world in the coming years due to the aging of the population, especially in high-income countries, and increased retention of natural teeth [4]. Nazir et al. reported disparities in the severity of periodontal disease among countries, where high-income countries had the highest prevalence of pocket depth [5]. Another study assessed the prevalence of plaque-induced gingivitis and found that 100% of 385 adult subjects aged between 18 and 40 years old had gingivitis [6]. Overall, about 20-50% of the population around the world has periodontal disease [2] with the most severe form affecting 11.2% of the world's population [3].

The systemic immune response might be influenced by periodontal pathogens as well as their metabolic by-products [7]. Advanced alveolar bone loss during periodontal infection is due to dysregulated inflammation and/or immunopathology, loosely reminiscent of the pathogenic mechanisms underlying certain systemic conditions [8]. Studies have reported a relationship between periodontal disease and a wide range of comorbidities including cardiovascular disorders (CVD), hypertension (HTN), diabetes mellitus (DM), rheumatoid arthritis, osteoporosis, Parkinson's disease, Alzheimer's disease, respiratory infections, and psoriasis [9]. In addition, the severity of periodontitis was linked with multiple comorbidities including gender, smoking, alcohol consumption, and pulmonary, endocrinal, metabolic, cardiovascular, neurological, hematological, and skeletal disorders [10]. Interestingly, it was found that individuals who had periodontal disease have a higher susceptibility for systemic comorbidities [11]. The majority of periodontitis cases exist in association with comorbidities including allergies, HTN, hyperlipidemia, and endocrine, pulmonary, musculoskeletal, and neurological disorders [12].

Over recent decades, there has been an increase in the global prevalence of DM [13]. Nearly, 451 million individuals have DM worldwide in 2017 [14]. In Saudi Arabia, DM is highly prevalent among the population, which represents a serious public health problem [15]. There is a bidirectional relationship between periodontitis and DM. DM augments periodontitis risks, and contrariwise, the inflammation in periodontal tissues negatively distresses glycemic control [7]. A recent observational study found that periodontitis is more prevalent in diabetic people than nondiabetic ones, with no difference in terms of gender and age [16]. Another study found that patients with DM type 2 and severe periodontal disease might counter higher mortality risk (3.2 times) as compared to no or mild periodontitis [2]. Moreover, the incidences of gingivitis and periodontitis were 21% and 6%, respectively, in type 1 diabetic children and adolescents [7]. On the other hand, there was a global increase in HTN prevalence of 5.2% over 10 years [17]. In Saudi Arabia, there are no current, accurate population-based estimates regarding the prevalence of HTN. Studies demonstrated that patients with periodontitis have higher systolic and diastolic pressures [18]. In 2010, the association between blood pressure values and periodontitis was examined in a large study, and the results showed a linear positive correlation [19]. The association between periodontitis and CVD was reported in several epidemiological investigations [7]. Studies have found that CVD risks could be 19% increased by periodontal diseases, whereas the risks might extend to 44% in elderly patients over 65 years [2]. The association between periodontitis and coronary heart disease (CHD) risks is independent of other risks such as smoking, DM, and socioeconomic status [2].

A variety of systemic and environmental risk factors may increase the prevalence and severity of chronic periodontitis. In addition, periodontal diseases may influence the pathogenesis of several systemic conditions such as CVD [20], DM [21], oral and colorectal cancers [22], and gastrointestinal [23] and respiratory diseases [24]. Recognizing the prevalence of multiple comorbidities in dental patients, especially with periodontitis, is a clinically important aspect that affects the patient's treatment protocol as well as its implications for public health strategy, guidelines, and health care worker training. Thus, the aim of our study was to assess the association between multiple comorbidity models and periodontal disease in a Saudi population from the Eastern province using regression analysis.

## 2. Materials and Methods

Participants eligible for this cross-sectional study were adult patients (>18 years old), who attended the periodontology clinic from September 2018 through September 2020 at the College of Dentistry at Imam Abdulrahman Bin Faisal University (IAU, Saudi Arabia). The current study was conducted in accordance with the Strengthening the Reporting of Observational Studies in Epidemiology (STROBE) guidelines. Exclusion criteria were individuals who underwent periodontal treatment over the last three months or were under continuous use of anti-inflammatory drugs, the presence of less than 12 teeth, malignancy, pregnancy, breastfeeding, and antibiotic use within 3 months prior to the study.

The study protocol was approved by the institutional review board, IAU (IRB-2021-02-034). Eligible participants were informed about the aims of the research and signed informed consent prior to entry into the study. Then, scheduled appointments were given for complete periodontal examination by two precalibrated examiners.

Patients' information regarding gender; age; nationality; medical history; use of medications; current systemic diseases, e.g., DM, HTN, hyperlipidemia, and CVD; and smoking habits (presence/absence) was collected. The fasting blood glucose level was measured, and serum levels of haemoglobin A1c (HbA1c) were recorded. DM was defined as *HbA*1*c* ≥ 6.5% (≥47.5 mmol/mol) or *FPG* ≥ 7.0 mmol/L. Systolic and diastolic blood pressures (SBP and DBP, respectively) were measured using an automatic blood pressure monitor. HTN was defined as *SBP* ≥ 140 mmHg or *DBP* ≥ 90 mmHg. Serum concentrations of triglyceride, high-density lipoprotein (HDL) cholesterol, low-density lipoprotein (LDL), and cholesterol were collected from recent medical records. Abnormal serum lipid levels were defined as *triglyceride* ≥ 150 mg/dL and/or HDL *cholesterol* < 40 mg/dL. Then, patients were further categorized depending on the presence of one or more comorbidity having DM and/or HTN as a main common disease.

Periodontal clinical examination was performed for all teeth except third molars, teeth with extensive carious lesions hindering the cementum-enamel junction (CEJ) determination, teeth with iatrogenic restorative procedures preventing the completion of the exam, teeth with Class 3 mobility, and unrestorable teeth indicated for extraction [25]. The following periodontal parameters were evaluated in all teeth present at six sites (mesiobuccal, distobuccal, buccal, lingual, mesiolingual, and distolingual) using a manual periodontal probe (UNC-15, Hu-Friedy, Chicago, IL), clinical mirror, and gauze. Clinical parameters included (1) probing depth (PD); (2) clinical attachment level (CAL); (3) bleeding on probing (BOP) which was assessed and categorized into <10%, 11–30%, and >30%; (4) number of missing teeth due to periodontal disease which was assessed and grouped as 0-2, 3-5, and >5 teeth; and (5) radiographic bone loss (RBL) which was measured on standardized bitewings and periapical radiographs that were done recently at the time of examination. RBL was calculated as the distance between the CEJ and the alveolar bone crest subtracted by 2 mm.

Severity of periodontal disease was reported using the suggested 2017 World Workshop Periodontal diseases and conditions [26]. Periodontitis was defined as having more than 2 detectable interproximal CAL, mild stage I periodontitis: the greatest interproximal CAL = 1–2 mm and *RBL* < 15%; moderate: stage II periodontitis: CAL = 3–4 mm and RBL = 15-33%; and severe periodontitis: stages III and IV without/with potential of edentulism (CAL ≥ 5 mm and RBL ≥ 30% or RBL ≥ 50%). Periodontal examinations were conducted by two precalibrated examiners. Intra- and interexaminer agreements were carried out on 20 individuals. Kappa values for PD and CAL proved to be higher than 0.90.

Statistical analysis was carried out using the statistical package for the social sciences (SPSS for Mac OS X, version 20.0, Inc., Chicago, IL, USA). Descriptive statistics were displayed as the mean ± standard deviation for quantitative variables, while frequencies and percentages were used for qualitative variables. We evaluated the normality of our data using the Kolmogorov-Smirnov test. The Mantel-Haenszel test of trend and Monte Carlo test were used to check association between comorbidities and severity of periodontal disease. Four main multivariate regression models were calculated, where CAL was the dependent variable in two of them and alveolar bone loss in the other two. Those models were aimed at classifying the individuals based on the presence of comorbidities, either DM alone or with the presence of other comorbidities (HTN, hyperlipidemia, or CHD), and its association with CAL and alveolar bone loss in 2 models. The other two models assessed the association between the presence of HTN alone and with other comorbidities (hyperlipidemia or CHD) and CAL and alveolar bone loss. All reported *P* values were considered statistically significant if less than 0.05.

## 3. Results

### 3.1. Characteristics of the Study Sample

**Figure 1:**
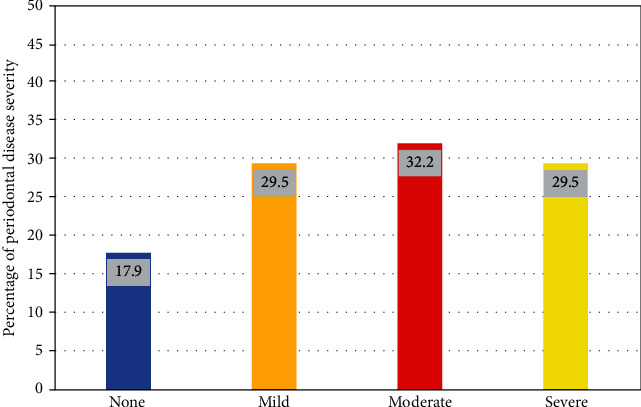
Severity of periodontal disease among participants.

Among 300 patients examined, the data of 190 patients (more than half of them aged 25-50 years; 134 females and 56 male) were included in the study. Exclusion criteria for the 110 subjects were subjects who underwent periodontal surgery within the last three months, patients under continuous use of anti-inflammatory drugs, patients under chemo- or radiotherapy, the presence of less than 12 teeth, pregnant and breastfeeding female patients, and subjects that did not show up in their scheduled appointment.  Table 1 shows the demographic data and oral health characteristics of the participants. The most common comorbidities found were DM (16.3%), followed by HTN (15.3%). Almost half of the patients had more than 5 teeth missing (49.5%). The mean pocket depth was found to be 3.6 ± 1.4, mean alveolar bone loss was 3.2 ± 3.8, and mean clinical attachment loss was 3.09 ± 2.58. The severity of periodontal disease among participants is presented in Figure 1; almost 30% of the participants had moderate periodontitis.

### 3.2. Results of Linear Regression Analysis between Each Variable

**Figure 2:**
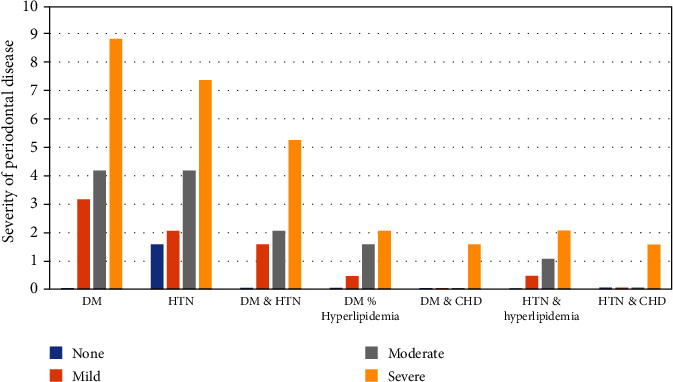
Frequency of multiple comorbidities according to the severity of periodontal disease. ^∗^Significant at *P* ≤ 0.05. ^$^Test of significance: Mantel-Haenszel test of trend with DM: ^∗^*P* < 0.001; HTN: ^∗^*P* = 0.008, and DM & HTN: ^∗^*P* = 0.002. ^†^Test of significance: Monte Carlo test used with DM & hyperlipidemia: *P* = 0.052, DM & CHD: ^∗^*P* = 0.03, HTN & hyperlipidemia: *P* = 0.051, and HTN & CHD: ^∗^*P* = 0.03.

Results of association between frequency of multiple comorbidities and severity of periodontal disease are shown in Figure 2. There was a statistically significant linear association between the presence of DM alone and HTN alone with the severity of periodontal disease (*P* < 0.001, 0.008, respectively). Also, there were significant associations when DM was combined with HTN or with CHD (*P* = 0.002, 0.03, respectively) and when HTN was combined with CHD (*P* = 0.03).

### 3.3. Linear Regression Analysis with Clinical Attachment Loss as the Dependent Variable

Tables 2 and 3 present the results of the linear regression analysis with CAL as the dependent variable.  Table 2 shows that the risk of CAL was significantly higher in persons whose age ranged from 25 to 50 years in DM, DM and HTN, DM and hyperlipidemia, and DM and CHD models (*B* = 1.43, 95%*CI* = 0.07, 2.76; *B* = 1.51, 95%*CI* = 0.13, 2.84; *B* = 1.53, 95%*CI* = 0.21, 2.90; and *B* = 1.52, 95%*CI* = 0.14, 2.95, respectively). Also, the risk of CAL was found to be statistically higher in patients who had 3 to 5 missing teeth in the 4 models (*B* = 1.18, 95% CI =0.09, 2.60; *B* = 1.15, 95%*CI* = 0.16, 2.75; *B* = 1.16, 95%*CI* = 0.14, 2.76; and *B* = 1.22, 95%*CI* = 0.22, 3.22, respectively). As for the comorbidities, patients suffering either from DM alone or from DM and HTN had significantly higher risk for CAL (*B* = 1.88, 95%*CI* = 0.43, 3.40, and *B* = 2.01, 95%*CI* = 0.49, 3.75, respectively). It was also higher in individuals with DM and hyperlipidemia or DM and CHD (*B* = 1.78, 95%*CI* = −2.24, 3.01, and *B* = 0.96, 95%*CI* = −3.04, 4.82, respectively); however, these associations were not statistically significant.  Table 3 also shows that HTN alone or with hyperlipidemia or CHD had higher risk for CAL; however, this was not statistically significant. On the other hand, patients whose ages were between 25 and 50 and those who had 3 to 5 missing teeth had significantly higher risk of CAL in the 3 models.

### 3.4. Linear Regression Analysis with Alveolar Bone Loss as the Dependent Variable

Tables 4 and 5 present the results of the linear regression analysis with average alveolar bone loss as the dependent variable. In Table 4, DM was the main comorbidity assessed whether alone or combined with other comorbidities. The risk of alveolar bone loss was higher in persons who had DM (*B* = 1.86, 95%*CI* = 0.30, 3.82) and those that had both DM and HTN (*B* = 2.82, 95%*CI* = 0.89, 4.75), with significant differences. The risk of alveolar bone loss was also higher among persons with DM and hyperlipidemia (*B* = 0.39, 95%*CI* = −2.24, 3.01) or CHD (*B* = 2.86 and 95%*CI* = −1.25, 6.96), but these differences were not statistically significant. For other independent variables, age and number of missing teeth were significantly associated with alveolar bone loss, where patients who were older than >25 years or had 3 to 5 missing teeth had higher risk of alveolar bone loss in all the 4 DM models. HTN was the main comorbidity assessed in Table 5, whether alone or with other comorbidities, where patients with HTN had significantly higher risk for alveolar bone loss (*B* = 1.67, 95%*CI* = 0.15, 3.18). In addition, age and number of missing teeth were significantly associated with alveolar bone loss.

## 4. Discussion

The association between periodontitis and immunomediated inflammatory disorders and comorbidities including HTN, type 2 DM, osteoporosis, hyperlipidemia, rheumatoid arthritis, and psoriasis has been extensively studied [9, 27, 28]. In our study, the severity of PD was shown to increase by the presence of one or more comorbidity. The Paksoy et al. study demonstrated that periodontitis severity was linked with multiple comorbidities including pulmonary, endocrinal, metabolic, cardiovascular, neurological, haematological, and skeletal disorders [10]. Similarly, our findings showed a linear pattern regarding the severity of periodontal disease in relation to the presence of one or more comorbidity presented in different models.

Our results showed that patients with DM (model 1), DM and HTN (model 2), and DM and hyperlipidemia (model 3) had 1.88, 2.01, and 1.78 times more probability to have deeper CAL than normal patients. Nevertheless, in HTN models, patients who had both HTN and hyperlipidemia presented 2.12 times more chance to develop deep CAL. Similarly, Mendes et al. [27] observed that psoriasis patients had 1.72 times more chance to present periodontitis as well as having deeper pockets than controls. Zhao et al. [11] observed that individuals with periodontal disease have higher susceptibility for systemic comorbidities after examining almost 500 records. Likewise, Lee et al. [29] collected data from 149,785 adults; they concluded that higher risk of periodontal disease could be predicted by a greater value of the Charlson comorbidity index especially in Korean patients above 60 years old.

Sperr et al. [12] conducted a study to evaluate 1199 Austrian individuals with periodontitis; they observed that majority of periodontitis cases are having comorbidities including allergies, HTN, and hyperlipidemia. Existing evidence showed that the local inflammatory response triggered in the periodontal tissue has systemic effects on inflammatory markers that negatively affect the cardiovascular system [30–32]. Thus, many cardiovascular risk factors and interrelated diseases, as well as HTN [33, 34] and atherosclerosis [35, 36], have been correlated with periodontitis. Moreover, previous studies showed that some periodontopathic bacteria are capable of inducing immune response activation and triggering neutrophil chemotaxis, thus inducing inflammation at remote sites. Similarly, the inflammatory response in periodontitis and other comorbidities is almost purely of neutrophilic nature [37, 38].

In the current study, 30% of the periodontitis patients reported one or multiple comorbidities. Peacock [39] observed that 52% of periodontal patients had systemic diseases. The difference could be due to the small sample size that was included in our study. Georgiou et al. [40] observed that almost 60% of periodontal patients are suffering from at least one comorbidity. They also observed that the prevalence of multiple medical conditions was higher in patients visiting periodontologists compared with patients in general practices. Georgiou et al. [40] reported also that periodontitis patients from all the three studied age groups (20-39, 40-59, and 60-79 years old) had a higher prevalence of DM. Similar to our findings, DM was also the most prevalent comorbidity reaching 16% among the examined patients.

Almost 60% of our study sample was in the middle age group (26-50 years), and 70% were female. The middle age (25-50 y) patients who had DM and/or HTN showed 1.4 times more CAL and 1.5 times more RBL, while patients older than 50 y showed almost 1.4 times more CAL and two times more RBL regardless of suffering from DM and/or HTN.

Many previous reports showed that the prevalence of systemic conditions increased with increasing age in both patients treated by a general practitioner vs. periodontist [39, 40]. A previous study on systemic disease prevalence of elder patients has shown that 64% of the candidates have at least one systemic disease [41]. Studies on periodontitis patients found that 47% of them reported having a systemic disease. However, as age increased, a steady increase was found in the percentage of systemic conditions reported. The frequency of systemic disorders in these patients increased from 21.1 percent in the youngest age group to 76.9 percent in the oldest age group [39, 42].

Tooth loss is considered the final sequel of untreated periodontal and dental diseases. It strongly affects the quality of oral health as well as influence the patient's quality of life [25]. In the present study, individuals with severe periodontitis coupled with DM and HTN presented a significantly higher number of missing teeth than the controls and remained as so in the final multivariate model. Similar results were reported in previous studies, in which patients suffering from psoriasis and periodontitis showed more tooth loss than control [25, 43, 44].

In our study, smoker patients showed more CAL and RBL in all comorbidity models than nonsmokers. Smoking, which is an environmental factor, could affect periodontal disease progression in adolescents [45–47]. Smoking promotes the destructive effect of inflammation in periodontitis [48]. Furthermore, smoking tobacco increases the risk for many other oral health problems, including oral cancer and CHD, and mortality [49].

A limitation of the current study was the relatively small number of patients; thus, this could affect the prevalence of some of the reported comorbidities. Thus, future studies containing a bigger sample are highly recommended. Also, prospective clinical studies are required to provide further clarification regarding the influence of periodontal treatment on the patient's systemic health.

## 5. Conclusions

There appears to be a positive association between multiple comorbidities and periodontal disease severity in terms of increased attachment loss, bone loss, and increased number of missing teeth.

## Figures and Tables

**Table 1:** Sample demographics and oral health characteristics (*N* = 190).

Variables		Frequency, *n* (%)
Age	18-25 years	30 (15.8)
	26-50 years	112 (58.9)
	>50 years	48 (25.3)

Gender	Males	56 (29.5)
	Females	134 (70.5)

Nationality	Saudi	61 (67.9)
	Non-Saudi	60 (32.1)

Smoking	Yes	33 (17.4)
	No	157 (82.6)

Comorbidities	DM	31 (16.3)
	HTN	29 (15.3)
	DM & HTN	17 (8.9)
	DM & hyperlipidemia	8 (4.2)
	DM & CHD	3 (1.6)
	HTN & hyperlipidemia	7 (3.7)
	DM & CHD	3 (1.6)

No. of missing teeth	0 to 2	15 (7.9)
	3-5	81 (42.6)
	>5	94 (49.5)

BOP (%)	1%-10%	43 (22.6)
	11-30%	95 (50.0)
	>30%	52 (27.4)

Pocket depth (mean ± SD)		3.6 ± 1.4

Amount of bone loss (mean ± SD)		3.2 ± 3.8

Clinical attachment loss (mean ± SD)		3.09 ± 2.58

**Table 2:** Association between CAL and DM with other comorbidities.

Factor		Model 1 DM		Model 2 DM & HTN		Model 3 DM & hyperlipidemia		Model 4 DM & CHD	
		*B* (95% CI)	*P* value	*B* (95% CI)	*P* value	*B* (95% CI)	*P* value	*B* (95% CI)	*P* value
Age	>50	1.05 (-0.97, 2.49)	0.34	1.36 (-0.53, 3.11)	0.14	1.47 (-0.35, 3.17)	0.08	1.76 (-0.13, 3.42)	0.07
	25-50	1.43 (0.07, 2.76)	0.04^∗^	1.51 (0.13, 2.84)	0.03^∗^	1.53 (0.21, 2.90)	0.03^∗^	1.52 (0.14, 2.95)	0.03^∗^
	18-<25	Reference		Reference		Reference		Reference	

Gender	Males	0.73 (-2.09, 0.63)	0.29	0.57 (-1.94, 0.81)	0.42	0.57 (-1.92, 0.81)	0.41	0.47 (-1.86, 0.91)	0.50
	Females	Reference		Reference		Reference		Reference	

Nationality	Saudi	0.49 (-0.50, 1.57)	0.32	0.41 (-0.59, 1.42)	0.25	0.32 (-0.68, -1.32)	0.53	0.40 (-0.61, 1.41)	0.44
	Non-Saudi	Reference		Reference		Reference		Reference	

Smoking	Yes	0.30 (-1.10, 1.67)	0.67	0.39 (-1.07, 1.86)	0.59	0.31 (-1.12, 1.73)	0.67	0.14 (-1.28, 1.56)	0.85
	No	Reference		Reference		Reference		Reference	

Number of missing teeth	>5	0.89 (-2.66, 0.81)	0.33	0.66 (-2.45, 1.12)	0.47	0.51 (-2.29, 1.28)	0.58	0.61 (-2.34, 1.19)	0.52
	3-5	1.18 (0.09, 2.60)	0.02^∗^	1.15 (0.16, 2.75)	0.02^∗^	1.16 (0.14, 2.76)	0.02^∗^	1.22 (0.22, 3.22)	0.01^∗^
	0-2	Reference		Reference		Reference		Reference	

Comorbidity	Yes	1.88 (0.43, 3.40)	0.01^∗^	2.01 (0.49, 3.75)	0.04^∗^	1.78 (-2.24, 3.01)	0.11	0.96 (-3.04, 4.82)	0.81
	No	Reference		Reference		Reference		Reference	

Model 1: effect of DM on CALs with other confounders controlled, adjusted *R*^2^ = 0.08, ^∗^*P* value = 0.004. Model 2: effect of DM and HTN on CALs with other confounders controlled, adjusted *R*^2^ = 0.10, ^∗^*P* value = 0.01. Model 3: effect of DM and hyperlipidemia on CAL with other confounders controlled, adjusted *R*^2^ = 0.06, ^∗^*P* value = 0.01. Model 4: effect of DM and CHD on CAL with other confounders controlled, adjusted *R*^2^ = 0.05, ^∗^*P* value = 0.02.

**Table 3:** Association between CAL and HTN with other comorbidities.

Factor		Model 1 HTN		Model 2 HTN & hyperlipidemia		Model 3 HTN & CHD	
		*B* (95% CI)	*P* value	*B* (95% CI)	*P* value	*B* (95% CI)	*P* value
Age	>50	1.44 (-0.45, 0.32)	0.13	1.47 (-0.31, 3.16)	0.11	1.76 (-0.13, 3.42)	0.07
	25-50	1.48 (0.09, 2.83)	0.03^∗^	1.53 (0.16, 2.91)	0.02^∗^	1.52 (0.14, 2.95)	0.03^∗^
	18-<25	Reference		Reference		Reference	

Gender	Males	0.51 (-1.87, 0.87)	0.47	0.54 (-1.90, 0.83)	0.44	0.47 (-1.86, 0.91)	0.42
	Females	Reference		Reference		Reference	

Nationality	Saudi	0.42 (-0.55, 1.43)	0.41	0.33 (-0.67, 1.33)	0.52	0.40 (-0.61, 1.41)	0.73
	Non-Saudi	Reference		Reference		Reference	

Smoking	Yes	0.19 (-1.23, 1.62)	0.79	0.28 (-1.13, 1.70)	0.69	0.14 (-1.26, 1.54)	0.08
	No	Reference		Reference		Reference	

Number of missing teeth	>5	0.67 (-2.45, 1.13)	0.46	0.49 (-2.27, 1.29)	0.59	0.61 (-2.34, 1.19)	0.52
	3-5	1.22 (0.23, 3.02)	0.01^∗^	1.13 (0.12, 2.32)	0.02^∗^	1.21 (0.24, 3.22)	0.01^∗^
	0-2	Reference		Reference		Reference	

Comorbidity	Yes	0.83 (-0.84, 2.09)	0.40	2.12 (-0.44, 4.03)	0.10	0.58 (-3.35, 4.31)	0.80
	No	Reference		Reference		Reference	

Model 1: effect of HTN on CAL with other confounders controlled, adjusted *R*^2^ = 0.06, ^∗^*P* value = 0.02. Model 2: effect of HTN and hyperlipidemia on bone loss with other confounders controlled, adjusted *R*^2^ = 0.06, ^∗^*P* value = 0.01. Model 3: effect of HTN and CHD on CAL with other confounders controlled, adjusted *R*^2^ = 0.05, ^∗^*P* value = 0.03.

**Table 4:** Association between alveolar bone loss and DM with multiple comorbidities.

Factor		Model 1 DM		Model 2 DM & HTN		Model 3 DM & hyperlipidemia		Model 4 DM & CHD	
		*B* (95% CI)	*P* value	*B* (95% CI)	*P* value	*B* (95% CI)	*P* value	*B* (95% CI)	*P* value
Age	>50	2.39 (0.43, 4.35)	0.017^∗^	2.20 (0.41, 4.21)	0.02^∗^	-2.74 (0.81, 4.07)	0.005^∗^	2.65 (0.75, 4.43)	0.005^∗^
	25-50	1.54 (0.11, 2.97)	0.03^∗^	1.56 (0.14, 2.97)	0.03^∗^	1.59 (0.16, 3.02)	0.03^∗^	1.62 (0.16, 3.02)	0.03^∗^
	18-<25	Reference		Reference		Reference		Reference	

Gender	Males	0.81 (-0.30, 1.91)	0.15	0.51 (-0.91, 1.92)	0.48	0.79 (-0.32, 1.90)	0.16	0.65 (0.46, 1.76)	0.25
	Females	Reference		Reference		Reference		Reference	

Nationality	Saudi	0.15 (-1.72, 0.15)	0.78	0.14 (-0.99, 1.07)	0.79	0.09 (-1.63, -0.05)	0.87	0.18 (-0.1.67, -0.07)	0.73
	Non-Saudi	Reference		Reference		Reference		Reference	

Smoking	Yes	1.40 (-0.39, 1.78)	0.21	1.78 (0.62, 3.61)	0.02^∗^	1.29 (-0.43, 1.75)	0.23	1.32 (-0.29, 1.89)	0.06
	No	Reference		Reference		Reference		Reference	

Number of missing teeth	>5	0.69 (-1.64, 2.12)	0.80	0.78 (-1.56, 2.13)	0.76	0.60 (-1.48, 2.28)	0.67	0.57 (-1.44, 2.29)	0.65
	3-5	1.63 (-2.66, -0.58)	0.01^∗^	1.52 (0.06, 3.49)	0.02^∗^	1.64 (0.70, 3.59)	0.02^∗^	1.65 (0.69, 4.61)	0.02^∗^
	0-2	Reference		Reference		Reference		Reference	

Comorbidity	Yes	1.86 (0.30, 3.82)	0.01^∗^	2.82 (0.89, 4.75)	0.004^∗^	0.39 (-2.24, 3.01)	0.60	2.86 (-1.25, 6.96)	0.17
	No	Reference		Reference		Reference		Reference	

Model 1: effect of DM on amount of bone loss with other confounders controlled, adjusted *R*^2^ = 0.20, ^∗^*P* value < 0.001. Model 2: effect of DM and hypertension on bone loss with other confounders controlled, adjusted *R*^2^ = 0.22, ^∗^*P* value < 0.001. Model 3: effect of DM and hyperlipidemia on bone loss with other confounders controlled, adjusted *R*^2^ = 0.19, ^∗^*P* value < 0.001. Model 4: effect of DM and CHD on bone loss with other confounders controlled, adjusted *R*^2^ = 0.19, ^∗^*P* value < 0.001.

**Table 5:** Association between alveolar bone loss and HTN with other comorbidities.

Factor		Model 1 HTN		Model 2 HTN & hyperlipidemia		Model 3 HTN & CHD	
		*B* (95% CI)	*P* value	*B* (95% CI)	*P* value	*B* (95% CI)	*P* value
Age	>50	2.17 (0.29, 4.12)	0.03^∗^	2.72 (0.85, 4.60)	0.005^∗^	2.65 (0.79, 4.50)	0.005^∗^
	25-50	1.48 (0.05, 2.90)	0.04^∗^	1.59 (0.15, 3.02)	0.03^∗^	1.63 (0.18, 3.03)	0.02^∗^
	18-<25	Reference		Reference		Reference	

Gender	Males	0.59 (-0.83, 2.01)	0.41	0.71 (-0.73, 2.15)	0.34	0.59 (-0.85, 2.02)	0.42
	Females	Reference		Reference		Reference	

Nationality	Saudi	0.19 (-0.85, 1.23)	0.72	0.08 (-0.97, 1.13)	0.88	0.18 (-0.86, 1.23)	0.73
	Non-Saudi	Reference		Reference		Reference	

Smoking	Yes	1.44 (-0.39, 2.91)	0.05	1.30 (-0.20, 2.80)	0.09	1.32 (-0.16, 2.79)	0.08
	No	Reference		Reference		Reference	

Number of missing teeth	>5	0.75 (-1.61, 2.11)	0.79	0.68 (-1.47, 2.29)	0.67	0.42 (-1.44, 2.29)	0.65
	3-5	1.65 (0.28, 2.62)	0.02^∗^	1.63 (0.09, 3.58)	0.03^∗^	1.81 (0.69, 3.61)	0.02^∗^
	0-2	Reference		Reference		Reference	

Comorbidity	Yes	1.67 (0.15, 3.18)	0.03^∗^	0.31 (-2.40, 3.03)	0.82	2.96 (-1.05, 6.96)	0.15
	No	Reference		Reference		Reference	

Model 1: effect of HTN on amount of bone loss with other confounders controlled, adjusted *R*^2^ = 0.21, ^∗^*P* value < 0.001. Model 2: effect of HTN and hyperlipidemia on bone loss with other confounders controlled, adjusted *R*^2^ = 0.19, ^∗^*P* value < 0.001. Model 3: effect of HTN and CHD on bone loss with other confounders controlled, adjusted *R*^2^ = 0.20, ^∗^*P* value < 0.001.

## Data Availability

Data will be provided upon request from the corresponding author.
